# The effectiveness of creating an online life story book on persons with early dementia and their informal caregivers: a protocol of a randomized controlled trial

**DOI:** 10.1186/s12877-017-0471-y

**Published:** 2017-04-21

**Authors:** Teuntje R Elfrink, Sytse U Zuidema, Miriam Kunz, Gerben J Westerhof

**Affiliations:** 1 Department of Psychology, Health and Technology, University of Twente, P.O. Box 217, 7500 AE Enschede, The Netherlands; 2 Department of General Practice and Elderly Care Medicine, University of Groningen, University Medical Center Groningen, PO Box 30.001, 9700 RB Groningen, The Netherlands

**Keywords:** Dementia, Life story book, Reminiscence, Informal caregivers, Rct, Neuropsychiatric symptoms

## Abstract

**Background:**

Dementia has a high burden for patients, informal caregivers and society. Given changes in care systems, more persons with dementia will live longer at home. However, living at home (with dementia) with a good quality of life is not easy to achieve. Dementia is often accompanied by neuropsychiatric symptoms like apathy, agitation, depression, and anxiety, which have a negative impact on quality of life. Whereas cognitive deterioration can hardly be influenced, it is possible to reduce neuropsychiatric symptoms. As autobiographical memories remain intact for a relatively long time in dementia, reminiscence interventions can promote feelings of pleasure and trust. The Online Life Story Book (OLSB) allows to digitally share memories (stories, pictures, video or audio fragments). The main objective is to study the effects of the OLSB on neuropsychiatric symptoms. The study has four secondary objectives: 1) to study the effectiveness of the intervention on the burden and quality of life of the primary informal caregiver; 2) to provide a preliminary health-economic evaluation; 3) to study the (time to) nursing home admittance as a longer term effect; 4) to provide a process evaluation.

**Methods and design:**

A randomized controlled trial with individual randomization to one of two conditions is conducted: 1) intervention “Online Life Story Book”; 2) control condition (care as usual). Participants are persons with early dementia and their primary caregivers. In the intervention OLSB, a trained volunteer guides the participants through the process of putting together a timeline of their lives during 5 meetings within a period of 8-10 weeks. To assess the effects of the intervention on the primary outcome, neuropsychiatric symptoms, the Neuropsychiatric Inventory (NPI) will be assessed at three time points: before the intervention (baseline, T0), 3 months (T1) and 6 months (T2) post baseline.

**Discussion:**

When proven effective, the Online Life Story Book can be a valuable addition to the existing provision of care for persons with dementia and their informal caregivers.

**Trial registration:**

This study has been approved by the Twente Medical Ethics Committee under the file number p16-04 (Dutch Trial Register: NTR5939, date of registration: 14 March 2016).

## Background

At present, the number of people living with dementia worldwide is estimated at 47.5 million. This number will increase to 75.6 million in the coming 15 years. Dementia is among the top-5 with the highest burden of disease for persons over 65 years and it belongs to the diseases with the highest burden for informal caregivers. This burden includes physical, emotional and economic pressure. With costs over 604 billion US dollars it is one of the most costly diseases as well [1].

In the Netherlands, about two thirds of persons with dementia is estimated to live at home, but given the impact of the ageing population this number will increase. This is also in line with the preferences of persons with dementia and their informal caregivers [2]. However, living at home with a good quality of life is not easy to achieve.

Dementia is often accompanied by the presence of neuropsychiatric symptoms (NPS), like apathy, agitation, depression, anxiety, and delusions. A systematic review of studies on the course of NPS in community-dwelling patients with dementia found a cumulative prevalence of any neuropsychiatric symptom between 49% and 95% [3]. Delusions, agitation, aberrant motor behavior and apathy are the most common NPS [3]. Neuropsychiatric symptoms not only affect the quality of life of the patient [4], but also result in a higher burden of informal caregivers and a lower quality of their lives [4–6]. NPS are among the most important reasons for nursing home admittance, as they often make the care at home too burdensome [7]. Whereas it is still not possible to treat dementia, dementia care focuses mainly on maintaining quality of life and preventing psychosocial problems [8].

There is evidence that neuropsychiatric symptoms can be prevented or diminished by behavioral interventions [9]. However, it is important that such interventions fit the experiences and life world of persons with dementia well so that they can contribute to the needs of the persons with dementia and their informal caregivers [10].

The Online Life Story Book (OLSB) is such an intervention that nicely ties in with changes in care systems that promote persons with dementia to be living longer at home instead of moving to a nursing home. The Online Life Story Book has a different approach than most existing applications as it focuses on the unique life story rather than on specific complaints and symptoms. The OLSB builds on research on reminiscence [11]. Research has shown that remembering and reliving precious personal memories can create feelings of pleasure, familiarity, and assurance. Hence, reminiscence interventions are part of the Dutch standard for dementia care [12]. Personal memories belong to the autobiographical memory system, a part of memory that remains intact in dementia for a relatively long time [13, 14]. Most persons with early dementia are able to retrieve personal memories and share them. And even in later phases of the disease, they can still relive the positive feelings associated with precious memories. When the disease progresses and memory further deteriorates, it becomes more and more important to offer multisensory cues for memories in a structured way that fits the unique life story of the person with dementia. Under these conditions, reminiscence interventions can contribute to the psychosocial functioning of persons with dementia [15].

Several systematic reviews have shown that reminiscence activities can contribute to the mental health and quality of life of persons with dementia [15–18]. The use of life story books with personal memories that were constructed together with the person with dementia is especially promising [18]. Besides the recollection of personal memories, the collection of a person’s life story in a book adds to this [19].

The current study aims to test the effects of the OLSB in comparison to care as usual. The current study adds in three ways to the existing knowledge about the use of life story books for persons with dementia: 1) by assessing effects on NPS in the home situation; 2) by using technology; 3) and by employing volunteers in the intervention.

### Assessing effects in the home situation

The current project is one of the first to use life story books in the home situation. Informal caregivers are in need of meaningful activities that allow them to step out of the caring role [20]. Mutual reminiscence is such an activity that can also give rise to further activities in everyday life such as listening to music together, or cooking an all-time favorite recipe. This helps to decrease NPS and promote quality of life of the person with dementia. Moreover, it aids in reestablishing a more personal relation with the person with dementia. It diminishes the burden of the informal caregiver and improves his or her quality of life.

### Using technology

The current project is innovative as it assesses the use of life story books that have been created with modern information and communication technology. Technology plays an increasing role in dementia care, whether in domotica, remote care, or mobile apps [21]. This also fits the increasing competences of older persons to use computers and the internet [22]. About 85% of the Dutch persons between 65 and 75 years already use the computer and the internet at least once a week [23]. The use of technology has three major advantages [24]. First, technology makes it easier to document and retrieve personal memories that match the idiosyncrasies of individual life stories. Second, technology provides multimedia for the storage and retrieval of memories. Sound, music, photos, and movies can be easily added, besides anecdotes and verbal cues. Indirectly smell, taste, and touch can be used, for example by cooking a favorite recipe or doing preferred activities together. This becomes even more important when it becomes more difficult for the person with dementia to retrieve memories through verbal stimuli. Third, technology makes it possible to use the life story book in an interactive way. Informal caregivers and family members and friends can add new memories or remarks on memories that were especially vivid to them. Hereby, it is possible to update and adjust the life story book even when the dementia progresses. A recent review shows that the use of technology for reminiscence intervention is promising, but that there is still a lack of systematic studies in this field [24].

### Employing volunteers

Given the changes in healthcare systems around the world in which more people will live longer at home instead of moving to a nursing home, not only informal caregivers but also volunteers play an increasing role and are expected to participate more and more to provide care in everyday life. For care institutes the delivery of the intervention will become more efficient than care provided by a professional [25]. For older adults, an intervention delivered by a volunteer will be less stigmatizing than care by a healthcare professional such as a psychologist, in particular while volunteers provide a new contact with society [26]. It is well-known that volunteering supports both the volunteer’s mental health and well-being and that of the people they serve [27]. Volunteers like to contribute to projects with a concrete goal, limited time investment, and possibilities for training [28]. The current project suits both altruistic and self-oriented motivations of volunteers as it offers the possibility to create something of personal value to the person with dementia and the informal caregiver, whereas it also allows them to learn new competences in the training [29, 30]. The volunteer provides the necessary structure and the needed social and technological competences that serve to ease the task for the informal caregiver. As the intervention is based on reminiscence as a naturally occurring process in later life, volunteers can easily stimulate conversations about personal memories with the help of a well-defined protocol [11].

In summary, the primary objective of the study is to assess the effectiveness of the intervention “Online Life Story Book” on neuropsychiatric symptoms of persons with early dementia, in comparison to care as usual. The study has four secondary objectives: 1) to study the effectiveness of the intervention OLSB on the burden and quality of life of the primary informal caregiver; 2) to provide a preliminary health-economic evaluation by analysing the effects on the quality of life and care consumption of the person with early dementia; 3) to study the (time to) nursing home admittance as a longer term effect; 4) to provide a process evaluation.

## Methods and design

### Study design

A randomized controlled trial with individual randomization to one of two conditions is conducted:

1. The intervention condition: participants in the intervention “Online Life Story Book”
2. The control condition: participants receive care as usual.

**Fig. 1:**
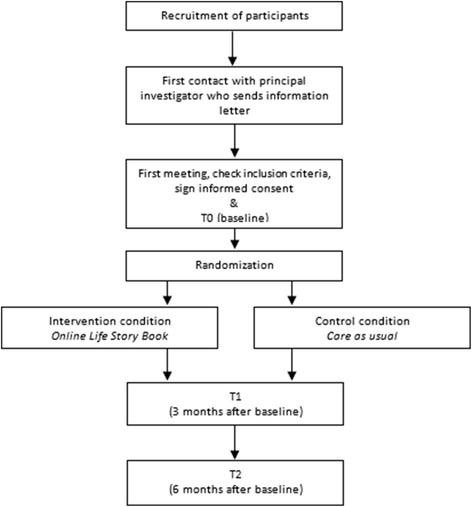
Study flowchart

The participant flow can be found in Fig. 1. The study takes 6 months for each individual participant with a baseline measurement (T0) as well as measurements after three (T1) and 6 months (T2). The intervention, creating an OLSB, takes about 8–10 weeks. The study takes place at the home of the person with early dementia who participates in the study. A trained volunteer will support the construction of the OLSB. Trained student assistants and the primary researcher will carry out the measurements.

This study has been approved by the Twente Medical Ethics Committee under the file number p16-04 (Dutch Trial Register: NTR5939). Participation is voluntary and all respondents will provide written informed consent before inclusion.

### Participants

#### Recruitment

We include persons with early dementia who are living at home and are being cared for by informal caregivers. In Enschede and Haaksbergen (two medium size cities in the Eastern part of the Netherlands) alone, there are 2500 persons with dementia. Livio, a large organization providing elderly care in this region, is in contact with about 575 of them through home care and living facilities for independently living older persons. Stichting Informele Zorg Twente, a local welfare organization, is in contact with about 650 informal caregivers. If the inclusion of 106 participants poses problems, participants can be recruited through other large care groups of the University Network for Elderly Care (UNO-UMCG), a network consisting of 16 large care organizations in the Northern and Eastern part of the Netherlands. We will recruit participants via so called Alzheimer Café’s, meeting centers for persons with early dementia, general practitioners and advertisements in newsletters for elderly people in the region.

#### Assessment of inclusion and exclusion

##### Inclusion and exclusion criteria

In order to be eligible to participate in this study, a subject must meet the following criteria:

- (Very) mild dementia;
- Living at home and receiving informal care;
- Being mentally capable to provide informed consent.

A potential subject who meets the following criterion will be excluded from participation in this study:

- Past psychotrauma.

##### Inclusion criteria

The main inclusion criterion is (very) mild dementia (Clinical Dementia Rating 0.5 or 1). This will be assessed with the Clinical Dementia Rating Scale [31]. This is a semi-structured interview that is carried out partly with the person with dementia and partly with the informal caregiver. The part for the person with dementia focuses on memory (10 items), orientation (8), and judgment/problem solving (9). The part for the caregiver asks for memory (15 items), orientation (8), judgment/problem solving (6), community affairs (10), home & hobbies (5), and personal care (4). The answers are used to compute a score ranging from 0 = none, 0.5 = very mild, 1 = mild, 2 = moderate to 3 = severe. Participants are included when they score 0.5 or 1. The other inclusion criteria are checked by the researcher during the baseline measurement.

##### Exclusion criterion

Psychotrauma is studied with the module posttraumatic stress disorder (PTSD) of the Mini International Neuropsychiatric Interview (MINI) that enables classification in DSM-categories [32]. The module consists of 14 dichotomous questions and 3 open questions that address current and lifetime PTSD. Participants are excluded when they report current or lifetime PTSD.

#### Sample size calculation

Based on systematic reviews [15, 17, 18], a small significant effect is expected for the primary outcome at follow-up. Hence, 74 participants are needed (GPower: f = 0.15; alpha = .05; power = .80; repeated measures anova with 2 groups and 3 measurement points; *r* = .50 between measurements). Given the vulnerability of the participants and a high mortality rate, we expect a drop-out of 30% [15, 17, 18]. Hence, 106 participants need to be included, 53 per condition.

### Randomization

After the participant is included in the study, signed the informed consent, and participated in the baseline measurement, randomization will be carried out. Randomization will be done by an independent researcher at the University of Twente, based on a computer-generated list of random numbers.

### Description of the intervention: the online life story book

The Online Life Story Book is an e-health application that allows placing personal memories on a dynamic timeline. The timeline is easily marked with historical years. Memories like life events, anecdotes, photos, movies, voice fragments, music, recipes, preferences, and activities can be placed on the timeline. To make the application safe and warrant privacy, all information is stored on a secured server. The application is developed by Hellomydear in Belgium [33].

#### OLSB materials

The application allows storage of materials that are directly relevant to the life story of the participant. Multimedia material can be used: text, photos, movies, sound, music, and indirectly smell, taste, touch, and movement, e.g., by adding recipes or preferences for activities. Subsequently, the participant and caregiver(s) can carry out these activities. The material may consist of personal information, such as photos of the participant at school, holidays, marriage, or with family and children. Material can also be added that bears a direct relation to the life story of the participants, such as an old picture of the street where someone lived, a logo of the firm for which the participant worked, a song or piece of music the participant liked. The subjective life story of the participant provides the necessary guidance. For a participant who liked football the history of his favorite club might be documented, whereas for a person who loved technology, reminders of technological innovation could be used. The material might be in possession of the participant or informal caregivers. Sometimes, it will be necessary to digitalize these materials, which is nowadays most easily done with the use of smartphones or tablets. Digital materials can also be found in other ways. Good websites document the social-cultural history of the Netherlands and/or its regions (e.g., [34–38]). Pictures or documents available for public use can be added to the timeline. The OLSB allows including links to these online materials if they are not available for public use.

#### Making the OLSB

Trained volunteers will support persons with dementia and their informal caregivers. After a first visit to make acquaintance and draw a global time line, the volunteer will visit the participant four additional times in 8–10 weeks. The volunteers follow guidelines that are based on existing scientific and practical insights into reminiscence and dementia [39, 40]. The volunteers are trained to ask for specific memories and to make them as explicit and lively as possible. Together with the person with dementia and his or her caregiver, the volunteer searches for the themes and questions that best match the life story of the participant. The volunteer strives for a variation in themes and life phases across the four visits. After each visit, the volunteer adds memories to the timeline. After the five visits and when the online life book is completed, it will be printed to a tangible version. We expect that it will take approximately 3 months from the first visit of the volunteer to the delivery of the printed book. After the initial construction of the online life book by the volunteer, it remains possible to add memories to the timeline as well as commentaries to memories. The participant will get access for the duration of 1 year. After this year, one gets the opportunity to lengthen the registration for €10,- per year. The necessary guidelines will be made available to the participants. The person with dementia and the caregiver can allow others, like children, families, or friends to contribute to the book.

#### Everyday use

The use of the OLSB in everyday life is very easy and pleasant. More competences than scrolling the timeline and clicking on memories is not needed. Besides the online version that is available on a computer or tablet, every participant will receive a printed version to make it easier to use in everyday life. Codes in the book make it possible to assess online material that cannot be printed, such as sound, music, or movies. Participants and their caregivers can make use of the online or printed version to recollect personal memories of the participant. Participants and caregivers receive a manual with tips and tricks how to use the application to this end and how they can make use of the memories in their everyday contact, for example by talking about favorite memories, listening to music, or doing preferred activities.

#### Time investment

A trained volunteer will aid the person with dementia in making the life-story book during five visits of 1 h. Based on existing reminiscence interventions [15, 17, 18], participants –and their informal caregiver and involved family– are advised to spend at least 1 h a week in using the book to recollect personal memories. Participants are instructed that this is a minimum requirement and that it is better to spend several shorter periods (e.g., 10-15 min several days a week) rather than 1 h on a separate day. Via the manual the participants also receive tips and tricks how to make use of the memories in planning everyday activities (e.g., listening to music, visiting the old primary school, baking ones favorite cookies, reading a rhyme). The time investment of these activities will vary depending on the kind of activities.

#### Control condition

Participants in the control condition receive care as usual. They have access to all existing treatments, interventions, and support, such as medical treatment, home care, memory centers, case management, activities organized by Alzheimer Nederland, support for informal caregivers. To allow for an optimal use of usual care, participants will be informed through a booklet about existing care possibilities. Participants in the control condition are allowed to create an OLSB after the study.

### Measurements

#### Overview

Table 1 gives an overview of all measurements. Participants are asked to fill out the questionnaires at baseline (T0); 3 months after the baseline measurement (T1); and 6 months after the baseline measurement (T2).

**Table 1:** Overview of study parameters, measurement instruments and measurement points

Measure	Instrument	Filled out by Person with dementia	Informal caregiver
*Inclusion criterion*			
*(Very) Mild dementia*	CDR	T0	T0
*Exclusion criterion*			
*Trauma*	MINI Psychiatric Interview, module PTSD	T0	
*Primary outcome measure*			
*Neuropsychiatric symptoms of person with dementia*	NPI, total score		T0, T1, T2
*Secondary outcome measures*			
*Caregiver burden*	NPI, module Distress		T0, T1, T2
	EDIZ		T0, T1, T2
	TOPICS-MDS, modules Time investment and Burden		T0, T1, T2
*Quality of life of caregiver*	TOPICS-MDS, modules CarerQol and Quality of life (RAND-36 and variant of Cantril’s Self Anchoring Ladder)		T0, T1, T2
*Health of caregiver*	TOPICS-MDS, module Health (RAND-36)		T0, T1, T2
*Health-economic evaluation*			
*Care consumption*	TOPICS-MDS, module Use of care		T0, T1, T2
*Health-related quality of life of person with dementia*	TOPICS-MDS, module EQ5D + Cognitive	T0, T1, T2	
*Personal Information*			
*Socio-demographics*	TOPICS-MDS, module General data		T0
*Personal functioning of person with dementia*	TOPICS-MDS, modules Daily functioning (Katz-15) and Social functioning (RAND-36)	Daily functioning: T0, T1, T2	Daily functioning & Social functioning: T0, T1, T2
*Health of person with dementia*	TOPICS-MDS, modules Health and Multimorbidity	Health: T0, T1, T2	Multimorbidity: T0, T1, T2
*Psychological well-being of person with dementia*	TOPICS-MDS, module Psychological well-being	T0, T1, T2	
*Quality of life of person with dementia*	TOPICS-MDS, module Quality of life (RAND-36 and variant of Cantril’s Self Anchoring Ladder)	T0, T1, T2	
*Longer term outcome*			
*Nursing home admittance*	Addressed in month 18 with general practitioner		
*Process evaluation*			
*Other (reminiscence) activities*	Open question		T1, T2
*Use of OLSB*	Open questions		T1, T2

#### Primary outcome

##### Neuropsychiatric symptoms (NPS)

To assess the effect of the intervention on the primary outcome, neuropsychiatric symptoms, the Neuropsychiatric Inventory (NPI) will be used at all three time points in all participants. The NPI is a reliable and valid measure that is filled out by the primary informal caregiver [41]. The NPI is often used to measure neuropsychiatric symptoms, also in pharmacological and psychosocial intervention studies. The instrument is developed by Cummings (1994) and translated in Dutch by De Jonghe, Borkend and Kat (1997) [42]. The NPI measures the frequency, severity and distress of twelve neuropsychiatric symptoms (Delusions, Hallucinations, Agitation/Aggression, Depression/Dysphoria, Anxiety, Elation/Euphoria, Apathy/Indifference, Disinhibition, Irritability/Lability, Aberrant motor behavior, Sleep and Nighttime Behavior Disorders, and Appetite and Eating Disorders). The frequency (F) is provided on a scale from 0 = never to 4 = daily, the severity (S) on a scale from 0 = not to 3 = severe. The score for each of the twelve symptoms is computed as the frequency multiplied by the severity, resulting in a score ranging from 0 to 12. An FxE score of 4 or higher is seen as clinically relevant symptoms. The twelve FxE scores are added to a total score (0-144) or to four scores of symptom clusters: hyperactivity, psychosis, affective symptoms, and apathy [43].

#### Secondary outcomes

The majority of the secondary outcomes and the personal information is assessed with parts of The Older Persons and Informal Caregivers Survey Minimum DataSet (TOPICS-MDS) [44]. The TOPICS-MDS was developed to examine the effects of the initiatives that are part of the Dutch National Care for the Elderly Programme.

##### Caregiver Burden

Caregiver burden is a secondary outcome measure. It is measured with a Dutch questionnaire (Ervaren Druk door Informele Zorg or EDIZ) [45]. This instrument has been used in informal caregivers with dementia and proved to be reliable and valid in this group. The informal caregiver rates the subjective burden on nine items with a five-point scale ranging from 1 = no! to 5 = yes!. The answers are dichotomized per item and then added up to a score between 0 and 9.

Caregiver burden is also measured with the distress scales of the NPI [41, 42]. The caregiver rates the distress for each of the twelve neuropsychiatric symptoms on a scale from 0 = none to 5 = severe. These twelve distress scores are summarized to a total score, ranging from 0 to 60. This sum score provides an indication of the emotional distress caused by all neuropsychiatric symptoms. Thirdly, the modules Time investment and Burden of the TOPICS-MDS are used to assess caregiver burden.

##### Quality of life of the caregiver

The care-related quality of life of the caregiver is measured with the CarerQol [46]. This reliable and valid instrument asks seven questions that are rated on a three-point scale (0 = none to 3 = many). Caregivers also answer a general question on happiness on a visual analogue scale ranging from 0 to 10. The total score provides an indication of the care related quality of life of the informal caregiver. The CarerQol is part of the TOPICS-MDS. In addition, the general quality of life of the caregiver is assessed with the module Quality of life of the TOPICS-MDS. The latter module consists of questions of the RAND-36 [47] and a variant of Cantril’s Self Anchoring Ladder [48] in which persons are asked to rate their life on a scale from 1 to 10.

##### Health of caregiver

The general health condition of the caregiver is measured with the module Health of the TOPICS-MDS. This consists of two questions of the RAND-36 [47].

#### Health-economic evaluation

*Care consumption* of the person with dementia will be used in the preliminary health-economic evaluation that provides a time window of 6 months, parallel to the RCT. Care consumption is measured in the TOPICS-MDS [44]. Care consumption includes medical costs (e.g., visits of general practitioner, specialist physicians, and hospital care) as well as indirect non-medical costs (e.g., home care, travel costs). Costs will be evaluated, based on the manual of Tan et al. (2012) [49].

##### Health-related quality of life of person with dementia

To measure health-related quality of life, the EQ5D + Cognitive will be used [50], a short questionnaire with 6 items that is also part of the TOPICS-MDS. This study will use Dutch tariffs to calculate the quality adjusted life years (QALYs) to obtain a utility score.

#### Personal information

##### Socio-demographics

The following socio-demographics of both the persons with dementia as well as their caregivers are assessed: sex, age, education, marital status and cultural background. These questions are part of the TOPICS-MDS, module General Data [44]. Additionally, the name of their general practitioner is also asked, so they can be informed about the participation in the study.

##### Personal functioning of person with dementia

Personal functioning is measured with the modules Daily functioning and Social functioning of the TOPICS-MDS. This module consists of the Katz-15-ADL [51] and a question of the RAND-36 [47].

##### Health of person with dementia

The general health condition and the multimorbidity are assessed with the modules Health and Multimorbidity of the TOPICS-MDS. The latter consists of a list of 17 common diseases.

##### Psychological well-being of person with dementia

The psychological well-being is measured with the module Psychological well-being of the TOPICS-MDS. This module consists of questions (5) of the RAND-36 [47].

##### Quality of life of person with dementia

The module Quality of life of the TOPICS-MDS is being used to measure the quality of life of the person with dementia. This module consists of two questions of the RAND-36 (2) about quality of life in general and at this moment [47] and a variant of Cantril’s Self Anchoring Ladder [48] in which persons are asked to rate their life on a scale from 1 to 10.

#### Longer term outcome

##### Nursing home admittance

The longer term outcome is (time to) admittance to a nursing home. This will be assessed for all participants in month 18 of the study together with the general practitioner.

#### Process evaluation

The intervention will be evaluated in two ways: a content analysis of Online Story Books and through qualitative interviews. Participants will be asked for renewed informed consent for this part of the study, after they constructed the Online Life Story Book, as they can only then judge whether or not they want to share it with the research team. A content analysis of twenty randomly chosen Online Life Story Books will be carried out.

Qualitative interviews will be conducted using an existing narrative method [52] after the study has been completed. Stakeholders (professionals (care manager, psychologist, coordinator of volunteer work, social worker), volunteers, participants and their informal caregivers) will be asked to provide stories about specific moments before, during, and after the intervention. Each interview will last about 1 h.

Next to the content analysis and interviews, at T1 and T2 participants are asked questions regarding:

##### Reminiscence Activities

In order to ascertain whether persons in the control group did not create an online life story book or have undertaken similar activities during the study, the informal caregivers are asked a question about which reminiscence activities the person with dementia has undertaken.

##### The use of the Online Life Story Book

Participants are asked how often and how long the book in general and the online version of the book is used.

### Statistical analyses

#### Descriptive statistics

First, a CONSORT flow diagram will be made that shows the flow of the participants as well as the number of participants and the reasons for dropping out of the intervention or the study. Descriptive analyses will provide insights in the major characteristics of the persons with early dementia and the informal caregivers that participated in the study. These include personal information and functioning (TOPICS-MDS), neuropsychological complaints (NPI), caregiver burden (NPI and EDIZ) as well as quality of life of the person with dementia (EQ5D + Cognitive) and the caregiver (CarerQOL). Comparison with existing data of the TOPICS-MDS will give more information about the group that was actually reached.

#### Randomization check

Chi-square tests and t-tests will be used to check whether the randomization has succeeded in terms of personal information and functioning (TOPICS-MDS), neuropsychological complaints (NPI), caregiver burden (NPI and EDIZ), quality of life of the person with dementia (EQ5D + Cognitive), and quality of life of the caregiver (CarerQOL). When significant differences are found at baseline, we will assess whether these attenuate the outcomes later in time. If that is the case, we will control for these confounding variables in later analyses on the effects of the intervention.

#### Missing values

Multiple imputation (five data sets) will be used to replace missing values and carry out the analyses on the pooled data set. Hence, all participants who were randomized can be included in the statistical analyses. The results from this imputed intention-to-treat sample will be compared to the results of the observed data only.

#### Effects

To analyze the effects on the primary outcome (total neuropsychiatric symptoms), repeated measures MANOVA will be used to assess the group differences across time. The condition (intervention vs. control) is the independent variable and the outcome measures across the three measurements are the repeated dependent variables. Simple contrasts will be used post-hoc to compare the post-intervention and follow-up measures with the pre-intervention measures. Effect sizes at post-intervention and follow-up will be calculated as Cohen’s d. Effect sizes between .56 and 1.2 are interpreted as large, between .33 and .55 as moderate, and below .33 as small [53].

We will also tentatively study the effects of the intervention on the four clusters of neuropsychiatric symptoms (hyperactivity, psychosis, affective symptoms, and apathy). As these measures are often not normally distributed, we will use a dichotomized outcome (clinically relevant symptoms or not, based on a FxS score of 4 and higher) [43]. We will carry out four logistic regression analyses with condition (intervention versus control) and presence of clinically relevant symptoms within the respective clusters at baseline as independent variables and presence of clinically relevant symptoms at follow-up as dependent variable. The odds-ratios will be used to provide estimates of the effect sizes in terms of Cohen’s d.

For the secondary outcome measures, three repeated measures MANOVA’s will be conducted, similar to the analysis for the primary outcome. The effect sizes will be given in Cohen’s d.

##### Health-economic evaluation

The preliminary health-economic evaluation will be done from a healthcare perspective. It is expected that the costs of the intervention will be earned back through the reduction in care consumption. The health-economic evaluation will be carried out according to the intention-to-treat principle. Bootstrapping techniques with 95% confidence intervals will be used to compare the differences in costs between the intervention and the control condition. The incremental cost-utility ratios (ICERs) will be computed. These indicate the extra costs per QALY (measured with the EQ5D + C) gained. A sensitivity analysis will be conducted to assess the robustness of the results by assessing the mean costs (plus or minus a standard deviation) for the most important expenses.

##### Nursing home admittance

The longer term effect of (time to) nursing home admittance will be analyzed by comparing the intervention and control condition in a Cox’s hazard regression analysis.

##### Process evaluation

A *content analysis* of twenty randomly chosen Online Life Story Books will be carried out. The analysis focuses on aspects of life stories and autobiographical memories that are known to be related to mental health [54]: the number of memories, the specificity of the memories (memories that refer to events that took place on a single day are considered specific memories), the variance of memories across life domains (family, friends, work, care, and household, leisure, health, historical events), the variance across life phases (childhood, adolescence, young adulthood, middle adulthood, older adulthood) and the variance across media (text, photos, movies, sounds, music). The codes will be quantified into a measure of the complexity of the Online Life Story Book. Descriptive statistics of the complexity measure as well as its correlation with change in neuropsychiatric symptoms between baseline and follow-up will be calculated.

*Qualitative interviews* will be analyzed through story line analysis [55]. Story line analysis allows assessing the most important personal values that are at stake in a story. A qualitative description of its results will be provided.

## Discussion

The first strength of this project is that the Online Life Story book is an intervention that builds on existing knowledge about reminiscence [11]. The last decade there is more evidence that reminiscence interventions are effective in improving cognitive functions and in decreasing feelings of depression in persons with dementia [16]. Life story books are often used as a tool to ease the process of recollecting autobiographical memories. However, little is known about the effects of using a life story book as reminiscence therapy for people with early dementia. We are conducting an RCT for persons with early dementia or mild cognitive impairment so that they can contribute to the book themselves. The OLSB utilizes the natural process of recollecting memories and stimulates this process. It is known that in an early stage of dementia, people can still retrieve and share memories from the past [13, 14]. However, our research focuses on a relatively short time period of 6 months. Some effects or changes, especially in care consumption, might only take place later in time, also because care institutions do not quickly adjust their provisions. An important question for future research is also how the competence to retrieve autobiographical memories changes over the course of the disease and how the book can be used to stimulate memory recollection when persons with dementia have less control over this process.

Another strong aspect of this project is that the OLSB intervention nicely ties in with changes in care systems promoting persons with dementia to be living at home for a longer period. Professional support will not follow this trend, so persons with dementia will depend more and more on informal caregivers and volunteers. We expect that the OLSB not only contributes to the NPS and quality of life of the person with dementia living at home, but also to the care burden and quality of life of informal caregivers. The current study mainly focuses on the effects for persons with dementia and their caregivers. Important questions for future research are how the intervention affects the well-being for volunteers and how the deployment of volunteers can be made sustainable when the intervention is implemented on a larger scale.

A third strength of the OLSB is the use of technology. Technology plays an important role in society –and dementia care– and the use of technology also fits the increasing competences of older persons to use computers and the internet [22]. People do not only receive a tangible book at the end but the lasting online exemplar will be available and gives the persons the opportunity to adjust its content as well as to use the multimedia sources to stimulate memory retrieval (e.g. watching a movie or listening to a specific song that is included in the book). Another advantage of the technology in the OLSB is the opportunity for nearest and dearest to give input and work along on the life story from a distance. Although we provide participants with a manual for the use of the OLSB, it will be important to monitor in future studies the actual use of the OLSB, for example by analyzing log data. The technology would also make it possible for professional caregivers to learn more about the life stories of the persons with dementia. This may support them in providing person-centered care. The OLSB can improve the relationship between care staff and persons with dementia and can contribute to fulfilling the needs of the client. These possibilities of technology ask for further research on how professionals should obtain access to the OLSB and how they can make use of the OLSB in their everyday care.

Most of the studies conducted in this research area consist of small samples [15, 16]. Our study is the first to carry out an RCT on an online life story book among persons with dementia living at home. The study closely follows everyday care practices in its recruitment. To reach the targeted number of participants it will be of great importance to work closely together with the partners to make sure that every potential channel for recruitment is being used. As the ‘online’ aspect might generate resistance among those who do not have much experience with computers, we stress that the participants receive a hard copy of the book and that they can leave the online tasks to the volunteer. People might also be reluctant to admit their cognitive complaints. We try to overcome this possible threshold by a positive recruitment strategy that focuses on the value and potential of the OLSB, rather than on the complaints. To gain insight in the recruitment procedures, we will keep track of the success of the recruitment. Furthermore, we will compare our sample to the Dutch population with dementia, based on data of the TOPICS-MDS, in order to gain more knowledge about the participants we reached. This is also important as the study is carried out in only one region (the eastern part of the Netherlands).

If proven to be effective, the Online Life Story Book may be offered as a standard intervention for persons with early dementia, living at home. As a follow up project, we want to address the implementation process of the Online Life Story Book in (care) practice.

## Abbreviations

CDR: Clinical Dementia Rating
EDIZ: Ervaren Druk door Informele Zorg
ICERs: Incremental cost-utility ratios
MINI: Mini International Neuropsychiatric Interview
NPI: Neuropsychiatric Inventory
NPS: Neuropsychiatric symptoms
OLSB: Online Life Story Book
PTSD: Posttraumatic stress disorder
QALYs: Quality adjusted life years
RCT: Randomized controlled trial
TOPICS-MDS: The Older Persons and Informal Caregivers Survey Minimum DataSet
UNO-UMCG: University Network for Elderly Care
